# Uptake and 24-month Outcomes of Dolutegravir- Versus Lopinavir-based Second-line Antiretroviral Therapy for People With HIV in South Africa: A Retrospective Cohort Study and Emulated Target Trial

**DOI:** 10.1093/ofid/ofaf530

**Published:** 2025-08-30

**Authors:** Jennifer Anne Brown, Lara Lewis, Yukteshwar Sookrajh, Lungile Hobe, Thulani Ngwenya, Johan van der Molen, Kwabena Asare, Kwena Tlhaku, Mlungisi Khanyile, Thokozani Khubone, Christian Bottomley, Nigel Garrett, Jienchi Dorward

**Affiliations:** Centre for the AIDS Programme of Research in South Africa (CAPRISA), University of KwaZulu-Natal, Durban, South Africa; Nuffield Department of Primary Care Health Sciences, University of Oxford, Oxford, UK; Centre for the AIDS Programme of Research in South Africa (CAPRISA), University of KwaZulu-Natal, Durban, South Africa; eThekwini Municipality Health Unit, eThekwini Municipality, Durban, South Africa; Mseleni Hospital, uMkhanyakude District, Nhlamvu, KwaZulu-Natal, South Africa; Bethesda Hospital, uMkhanyakude District, uBombo, KwaZulu-Natal, South Africa; Centre for the AIDS Programme of Research in South Africa (CAPRISA), University of KwaZulu-Natal, Durban, South Africa; Centre for the AIDS Programme of Research in South Africa (CAPRISA), University of KwaZulu-Natal, Durban, South Africa; Department of Non-Communicable Disease Epidemiology, Faculty of Epidemiology and Population Health, London School of Hygiene and Tropical Medicine, London, UK; Centre for the AIDS Programme of Research in South Africa (CAPRISA), University of KwaZulu-Natal, Durban, South Africa; Department of Public Health Medicine, School of Nursing and Public Health, University of KwaZulu-Natal, Durban, South Africa; Centre for the AIDS Programme of Research in South Africa (CAPRISA), University of KwaZulu-Natal, Durban, South Africa; eThekwini Municipality Health Unit, eThekwini Municipality, Durban, South Africa; Department of Infectious Disease Epidemiology and International Health, Faculty of Epidemiology and Population Health, London School of Hygiene and Tropical Medicine, London, UK; Centre for the AIDS Programme of Research in South Africa (CAPRISA), University of KwaZulu-Natal, Durban, South Africa; Department of Public Health Medicine, School of Nursing and Public Health, University of KwaZulu-Natal, Durban, South Africa; Desmond Tutu HIV Centre, University of Cape Town, Cape Town, South Africa; Centre for the AIDS Programme of Research in South Africa (CAPRISA), University of KwaZulu-Natal, Durban, South Africa; Nuffield Department of Primary Care Health Sciences, University of Oxford, Oxford, UK

**Keywords:** antiretroviral therapy, dolutegravir, retention in care, ritonavir-boosted lopinavir, viral load

## Abstract

**Background:**

Aligning with the World Health Organization, South Africa has replaced ritonavir-boosted lopinavir (LPV/r) with dolutegravir (DTG) in second-line antiretroviral therapy (ART) after treatment failure with tenofovir disoproxil fumarate (TDF)/lamivudine or emtricitabine (XTC)/efavirenz (EFV). Initial guidance included special considerations for DTG use among women.

**Methods:**

We analyzed routine deidentified data of adults switched from TDF/XTC/EFV to second-line AZT/XTC/LPV/r, AZT/XTC/DTG, or TDF/XTC/DTG between December 2019 and December 2023 at 108 healthcare facilities in KwaZulu-Natal, South Africa. Among people switched before July 2021, we emulated a target trial comparing 24-month death or loss to follow-up (LTFU), and viremia (>50 copies/mL). We conducted intention-to-treat and per-protocol analyses using weighted logistic regression with bootstrapped CIs.

**Results:**

Overall, women were less likely than men to switch to DTG (RR: 0.92 [95% CI: .88, .96]; *N* = 3649). Of 2321 people switched before July 2021, 915 (39%) switched to AZT/XTC/LPV/r, 415 (18%) to zidovudine (AZT)/XTC/DTG, and 991 (43%) to TDF/XTC/DTG. Median age was 36 years (IQR: 30, 43) and 1364 (59%) were women. In intention-to-treat analyses, the standardized 24-month risk of death or LTFU was similar with AZT/XTC/LPV/r (31%), AZT/XTC/DTG (30%), and TDF/XTC/DTG (34%). The standardized risk of 24-month viremia among those retained in care with a viral load result (*N* = 1270) was higher with AZT/XTC/LPV/r (50%) than with AZT/XTC/DTG (40%; aRD: −10% [95% CI −19%, −2%]) or TDF/XTC/DTG (39%; aRD: −11% [95% CI −18%, −5%]). Per-protocol analyses gave similar results.

**Conclusions:**

While retention was similar across regimens, viremia was less common on DTG-based ART, supporting current guidelines.

## Teaser

A target trial emulation including adults switched from TDF/XTC/EFV to second-line AZT/XTC/LPV/r, AZT/XTC/DTG, or TDF/XTC/DTG in a South African HIV cohort (*N* = 2321) showed similar 24-month outcomes of death or loss to follow-up but less viremia on DTG-based ART.

Since 2018, the World Health Organization has recommended antiretroviral therapy (ART) containing dolutegravir (DTG) as the preferred second-line regimen upon treatment failure with a regimen containing efavirenz (EFV), replacing ritonavir-boosted lopinavir (LPV/r) in second-line ART [1, 2]. This recommendation was informed by the DAWNING trial, which showed superior 48-week viral suppression with DTG- compared with LPV/r-based second-line ART [3]. The NADIA trial subsequently showed recycling tenofovir disoproxil fumarate (TDF) from first-line ART in second-line DTG-based regimens was noninferior to changing to zidovudine (AZT) at 48 and 96 weeks post switch [4, 5].

In South Africa, DTG was rolled out for use in first- and second-line ART in December 2019. In first-line ART, DTG was associated with better clinical outcomes than EFV as the former standard of care [6]. In the early stages of the rollout, women were less likely than men to receive first-line DTG due to initial safety concerns regarding DTG in early pregnancy, though this difference declined over time with newer evidence and updated guidelines recommending DTG-based ART also for adolescent girls and women of childbearing potential [1, 6]. However, there is little data regarding uptake of second-line DTG by gender. For second-line ART, previous analyses of routinely collected primary care data in South Africa showed superior 12-month retention in care with AZT/lamivudine or emtricitabine (XTC)/DTG compared with either AZT/XTC/LPV/r or TDF/XTC/DTG, and superior 12-month viral suppression with either DTG-based second-line regimen than with AZT/XTC/LPV/r [7].

While these data are encouraging, continued monitoring with longer-term follow-up in large, routine care datasets remains essential, especially in light of recent reports of emergent DTG resistance [8–10]. Therefore, we aimed to assess uptake of DTG-based ART by gender, and subsequent 24-month outcomes between second-line AZT/XTC/LPV/r, AZT/XTC/DTG, or TDF/XTC/DTG in routine care settings in South Africa. We hypothesized that DTG-based ART would lead to improved treatment outcomes compared with LPV/r.

## METHODS

### Study Design and Setting

We used deidentified, routinely collected data from South Africa's ART program. We included data from 108 public sector primary healthcare clinics in the eThekwini Municipality and uMgungundlovu district in KwaZulu-Natal province, South Africa in this analysis.

We created an uptake and an outcome cohort, with the latter having an earlier cutoff for regimen switches allowing sufficient time to ascertain outcomes. In the outcome cohort, we emulated a target trial to compare death and loss to follow-up (LTFU) through 24 months, and viremia at 24 months, by second-line regimen. The prespecified protocol of the target trial as well as key components of how it was emulated are described in Supplementary Table 1.

This study is reported according to the Strengthening the Reporting of Observational Studies in Epidemiology guidelines and in alignment with emerging recommendations on reporting target trial emulation [11–16].

### Participants

In the uptake cohort, we included people ≥15 years of age who switched from TDF/XTC/EFV to AZT/XTC/LPV/r, AZT/XTC/DTG, or TDF/XTC/DTG between December 1, 2019 (the start of DTG availability) and December 31, 2023 (data closure) following confirmed virological failure. Confirmed virological failure was defined as 2 consecutive viral loads ≥1000 copies/mL taken 56–450 days apart. We excluded people whose most recent virologic failure was >450 days before the switch and who transferred out on the day of the regimen switch. We also excluded people with known prior exposure to a protease or integrase strand transfer inhibitor or who were pregnant at the time of switch, as these factors might causally affect the choice of regimen and the outcomes yet numbers were insufficient to allow for adjustment.

In the outcome cohort, we applied the same eligibility criteria except that the switch had to occur by June 30, 2021, allowing 30 months for outcome ascertainment (see Supplementary Table 1 for rationale).

### Outcome Measures and Exposures

In the uptake cohort, the main outcome of interest was the risk of receiving DTG-based ART, ie, either of the DTG-containing regimens. The main exposure of interest was gender.

In the outcome cohort, the coprimary endpoints were (1) time to death or LTFU through 24 months after switch, and (2) the risk of 24-month viremia. The date of LTFU was defined as the midpoint between the last attended visit and the scheduled date of the first visit missed by at least 90 days. People who transferred out of their facility were censored at the date of transfer. Those remaining in care were censored at 24 months post switch. The viremia endpoint was defined as having a viral load >50 copies/mL 24 months (window: 18–30 months) after switch. If several viral load results were recorded within this window, we selected the result closest to 24 months. The main exposure of interest was the second-line regimen initiated at switch, ie, AZT/XTC/LPV/r, AZT/XTC/DTG, or TDF/XTC/DTG.

In sensitivity analyses, we assessed a composite of death, LTFU, and transfer-out, and defined 24-month viremia as ≥1000 copies/mL.

### Data Sources/Measurement

We used deidentified data from the South African Three Interlinked Electronic Register database (TIER.Net) [17, 18], a 3-tier monitoring system for ART, tuberculosis, and mother and child health services. TIER.Net covers all people receiving ART in public sector clinics. After every clinic visit, a data capturer enters relevant clinical information. For this analysis, we assessed demographic data, clinical status (including date of death or transfer-out), ART regimen, tuberculosis treatment, pregnancy, laboratory results (CD4 cell count and viral load), and clinic visit information (visit dates, scheduling, and referral to decentralized ART programs) of people receiving ART in public sector healthcare facilities.

### Statistical Methods

In the uptake cohort, we used Poisson regression with robust standard errors assuming an independent correlation structure to assess the relative risk (RR) of switching to DTG-based ART (binary outcome) by gender. We ran separate 3 separate models with (1) gender, (2) gender and age with an interaction term between gender and age, and (3) gender and calendar period (before or after June 2021, when South African guidelines recommended DTG for all) with an interaction term between gender and calendar period, as covariates (Supplementary Table 2). We tested for interaction using likelihood ratio tests.

To assess treatment outcomes by second-line ART regimen in the outcome cohort, we conducted intention-to-treat and per-protocol analyses for both coprimary endpoints.

First, we calculated inverse probability of treatment weights (IPTWs) to emulate randomization. To this end, we conducted multinomial logistic regression with the treatment as the outcome and baseline variables (listed in Supplementary Table 1) as covariates to derive propensity scores. Stabilized IPTWs were obtained by multiplying the inverse of the propensity scores by the proportion of participants receiving the respective treatment.

Second, for per-protocol analyses, we calculated inverse probability of censoring weights (IPCWs) to estimate outcomes that would have occurred without censoring. In per-protocol analyses, for the coprimary endpoint of death or LTFU, participants were censored for regimen change and transfer-out; for the sensitivity analysis with the endpoint of death, LTFU, or transfer-out, participants were censored for regimen change only; and for the coprimary and sensitivity endpoints of viremia, participants were censored for regimen change or nonretention (transfer-out, death, or LTFU). We ran pooled logistic regression models with censoring (as defined for the respective analysis) as the outcome, and treatment as well as baseline and time-varying variables (which could vary by month) as covariates. We used these models to predict monthly probability of censoring, from which we calculated the cumulative probability of remaining uncensored up to this time point. IPCWs were calculated as the inverse of the probability of remaining uncensored and stabilized by multiplying with corresponding probabilities obtained through models without time-varying covariates [19]. For the per-protocol analysis of 24-month viremia, we also calculated inverse probability weights of having a 24-month viral load result. To obtain stabilized weights, we multiplied the inverse probability of having a 24-month viral load obtained from logistic regression using treatment and baseline variables as covariates by the overall proportion of participants with a 24-month viral load result. The stabilized IPTWs, IPCWs, and inverse probability weights for having a 24-month viral load result were all truncated at the first and 99th centile. Further information on the covariates included in each model is available in Supplementary Table 1.

In intention-to-treat analysis for the coprimary endpoint of death or LTFU, we estimated hazard ratios using pooled logistic regression with treatment as the exposure of interest and time (months and months squared, each with an interaction term with treatment) and baseline variables as covariates, weighted with IPTW. Using this model, we derived the standardized monthly hazards of death or LTFU for each treatment assuming all participants received the treatment. We then calculated the standardized 24-month risk for each treatment from the monthly hazards through 24 months. Per-protocol analysis was the same, except that we weighted by the product of the IPTWs and IPCWs.

Viremia was assessed in intention-to-treat analysis through logistic regression with treatment as the exposure and baseline variables as covariates, weighted with IPTW. Implicitly, this analysis estimates viremia among people who were retained in care and had a 24-month viral load result. By contrast, the per-protocol analysis was weighted with IPTWs, IPCWs at 24 months (derived from longitudinal data), and the inverse probability of having a 24-month viral load result. Implicitly, this analysis aims to estimate viremia among all participants had they remained in care with viral load outcome data.

Sensitivity analyses were conducted as described for the primary endpoints. 95% confidence intervals (CIs) were generated using 500 bootstrap samples.

For descriptive outcomes, we report categorical variables with frequencies and percentages, and continuous variables with medians and interquartile ranges (IQRs). For inferential outcomes, we report standardized risks, adjusted risk differences (aRDs), and RRs with 95% CIs.

Analyses were conducted in R version 4.4.0.

## RESULTS

### Participant Characteristics in the Uptake Cohort

Between December 1, 2019 and December 31, 2023, 3814 people taking TDF/XTC/EFV switched to AZT/XTC/LPV/r, AZT/XTC/DTG, or TDF/XTC/DTG after virological failure. We excluded 165 participants who were <15 years old, had prior exposure to protease or integrase strand transfer inhibitors, were pregnant at switch, or transferred out on the day of the regimen switch. The remaining 3649 participants were included in the uptake cohort (Supplementary Figure 1); 998 (27%) switched to AZT/XTC/LPV/r, 711 (19%) to AZT/XTC/DTG, and 1940 (53%) TDF/XTC/DTG. 2292 (63%) were women, median age at switch was 36 years (IQR 29, 42), and median known time with viremia ≥1000 copies/mL was 397 days (IQR 251, 650; Supplementary Table 3).

### Observed Switches and Effect of Gender

**Figure 1.**
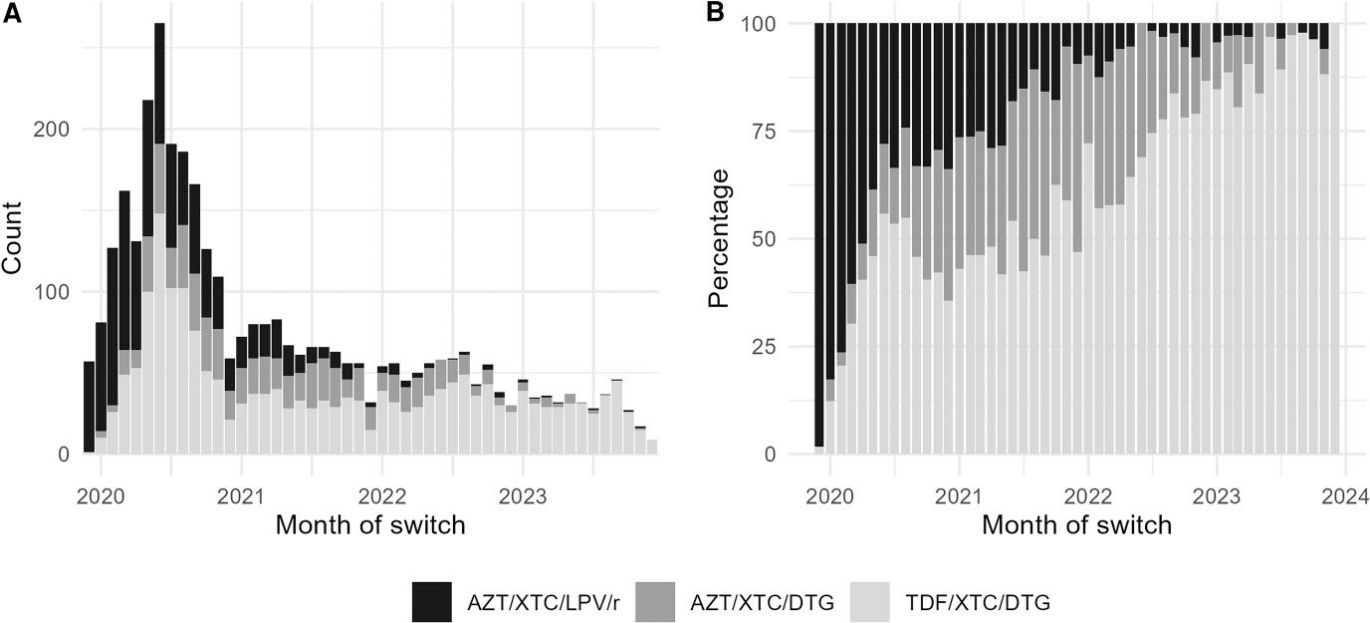
Switches to second-line ART over time. *A*. Number of switches to each second-line regimen per month. *B*. Percentage of switches to each second-line regimen per month. Abbreviations: ART, antiretroviral therapy; AZT, zidovudine; DTG, dolutegravir; LPV/r, ritonavir-boosted lopinavir; TDF, tenofovir disoproxil fumarate; XTC, lamivudine or emtricitabine.

The number of monthly switches increased from 57 in December 2019 to 265 in June 2020, before dropping to below 50 for all months after November 2022 (Figure 1*A*). AZT/XTC/LPV/r was the most frequently selected second-line regimen until May 2020, when TDF/XTC/DTG became the most-used regimen at switch (Figure 1*B*).

The RR for women (compared with men) to receive a DTG-based regimen was 0.92 (95% CI .88, .96). We found no evidence that the effect of gender on DTG use was modified by age category (likelihood ratio test *P* = .83; Table 1), whereas the RR for women to receive DTG was lower in December 2019–May 2021 (0.80; 95% CI .75, .85) and subsequently equalized in June 2021–December 2023 (0.97; 95% CI .95, 1.00; likelihood ratio test *P* = .013; Table 1).

**Table 1.** Relative Risk for Women to Switch to DTG-based ART in the Uptake Cohort

	Proportion of Men Switched to DTG	Proportion of Women Switched to DTG	RR (95% CI)
Overall	1038/1357 (76%)	1613/2292 (70%)	.92 (.88, .96)
By age^a^			
15–29 y	164/215 (76%)	513/702 (73%)	.96 (.88, 1.05)
30–44 y	589/782 (75%)	873/1267 (69%)	.91 (.87, .97)
≥45 y	285/360 (79%)	227/323 (70%)	.89 (.81, .97)
By calendar period^b^			
December 2019–May 2021	637/935 (68%)	719/1325 (54%)	.80 (.75, .85)
June 2021–December 2023	401/422 (95%)	894/967 (92%)	.97 (.95, 1.00)

RRs were assessed through Poisson regression with robust standard errors (*N* = 3649).

CI, confidence interval; DTG, dolutegravir; RR, relative risk.

^a^Likelihood ratio test for interaction term between gender and age: *P* = .83.

^b^Likelihood ratio test for interaction term between gender and calendar period: *P* = .013.

### Participant Characteristics in the Outcome Cohort

Of the 3649 participants in the uptake cohort, 2321 switched before June 30, 2021 and were included in the outcome cohort (Supplementary Figure 1). Of these, 915 (39%) switched to AZT/XTC/LPV/r, 415 (18%) to AZT/XTC/DTG, and 991 (43%) to TDF/XTC/DTG. Overall, 1364 (59%) were women, median age at switch was 36 years (IQR: 30, 43), and median known time with a viral load ≥1000 copies/mL was 371 days (IQR: 236, 581). After applying IPTWs, groups were balanced for baseline characteristics (Table 2).

**Table 2.** Baseline Characteristics in the Outcome Cohort and the Outcome Cohort Pseudo-population After IPTW

Characteristic	Outcome Cohort				Outcome Cohort Pseudo-population After IPTW			
	Overall *N* = 2321	AZT/XTC/LPV/r *N* = 915	AZT/XTC/DTG *N* = 415	TDF/XTC/DTG *N* = 991	Overall *N* = 2307	AZT/XTC/LPV/r *N* = 911	AZT/XTC/DTG *N* = 406	TDF/XTC/DTG *N* = 991
Women	1364 (59%)	614 (67%)	219 (53%)	531 (54%)	1353 (59%)	536 (59%)	239 (59%)	579 (58%)
Age in years	36 (30, 43)	36 (30, 42)	36 (30, 43)	36 (30, 43)	36 (30, 43)	36 (30, 43)	35 (30, 42)	36 (30, 43)
15–29	557 (24%)	221 (24%)	94 (23%)	242 (24%)	545 (24%)	210 (23%)	101 (25%)	234 (24%)
30–44	1325 (57%)	539 (59%)	238 (57%)	548 (55%)	1327 (58%)	527 (58%)	235 (58%)	565 (57%)
≥ 45	439 (19%)	155 (17%)	83 (20%)	201 (20%)	435 (19%)	174 (19%)	69 (17%)	192 (19%)
Receiving tuberculosis treatment	37 (1.6%)	14 (1.5%)	12 (2.9%)	11 (1.1%)	33 (1.4%)	14 (1.5%)	9 (2.2%)	11 (1.1%)
Region								
eThekwini Metropolitan Municipality	1304 (56%)	579 (63%)	243 (59%)	482 (49%)	1291 (56%)	508 (56%)	219 (54%)	564 (57%)
uMgungundlovu District Municipality	1017 (44%)	336 (37%)	172 (41%)	509 (51%)	1017 (44%)	403 (44%)	187 (46%)	427 (43%)
Years since ART initiation	3.23 (1.68, 6.03)	3.21 (1.63, 6.00)	3.39 (1.57, 6.69)	3.18 (1.75, 5.86)	3.26 (1.69, 5.98)	3.38 (1.70, 5.98)	3.53 (1.65, 6.52)	3.05 (1.69, 5.73)
CD4 cell count at ART initiation in cells/µL^a^	190 (100, 310)	170 (90, 290)	150 (80, 250)	220 (120, 340)	190 (100, 300)	170 (90, 290)	160 (90, 260)	210 (110, 330)
< 200	871 (38%)	385 (42%)	173 (42%)	313 (32%)	881 (38%)	373 (41%)	170 (42%)	337 (34%)
200–349	486 (21%)	185 (20%)	73 (18%)	228 (23%)	495 (21%)	194 (21%)	76 (19%)	224 (23%)
350–499	205 (8.8%)	75 (8.2%)	28 (6.7%)	102 (10%)	200 (8.7%)	70 (7.7%)	29 (7.2%)	100 (10%)
≥ 500	124 (5.3%)	38 (4.2%)	15 (3.6%)	71 (7.2%)	126 (5.5%)	43 (4.7%)	16 (4.0%)	67 (6.8%)
Missing	635 (27%)	232 (25%)	126 (30%)	277 (28%)	606 (26%)	231 (25%)	114 (28%)	261 (26%)
Days from CD4 count at ART initiation to ART initiation^a^	0 (0, 0)	0 (0, 0)	0 (0, 0)	0 (0, 0)	0 (0, 0)	0 (0, 0)	0 (0, 0)	0 (0, 0)
Missing	635 (27%)	232 (25%)	126 (30%)	277 (28%)	606 (26%)	231 (25%)	114 (28%)	261 (26%)
Most recent CD4 cell count in cells/µL^b^	260 (140, 420)	250 (130, 430)	230 (100, 380)	280 (165, 450)	260 (150, 430)	250 (140, 430)	260 (130, 420)	260 (160, 430)
< 200	575 (25%)	232 (25%)	133 (32%)	210 (21%)	549 (24%)	220 (24%)	98 (24%)	232 (23%)
200–349	447 (19%)	164 (18%)	84 (20%)	199 (20%)	459 (20%)	180 (20%)	83 (20%)	196 (20%)
350–499	296 (13%)	117 (13%)	50 (12%)	129 (13%)	284 (12%)	113 (12%)	53 (13%)	118 (12%)
≥ 500	283 (12%)	101 (11%)	44 (11%)	138 (14%)	273 (12%)	103 (11%)	44 (11%)	126 (13%)
Missing	720 (31%)	301 (33%)	104 (25%)	315 (32%)	743 (32%)	295 (32%)	128 (32%)	320 (32%)
Days from most recent CD4 result to switch	218 (58, 508)	228 (56, 586)	145 (47, 473)	234 (71, 495)	227 (61, 545)	231 (58, 605)	152 (56, 513)	236 (71, 508)
Missing	720 (31%)	301 (33%)	104 (25%)	315 (32%)	743 (32%)	295 (32%)	128 (32%)	320 (32%)
Prior exposure to AZT	24 (1.0%)	6 (0.7%)	7 (1.7%)	11 (1.1%)	26 (1.1%)	8 (0.9%)	6 (1.6%)	12 (1.2%)
Prior referral to decentralized ART	427 (18%)	152 (17%)	84 (20%)	191 (19%)	458 (20%)	175 (19%)	98 (24%)	185 (19%)
Last viral load in copies/mL								
1'000–9'999	917 (40%)	329 (36%)	132 (32%)	456 (46%)	908 (39%)	360 (40%)	162 (40%)	385 (39%)
10'000–99'999	969 (42%)	384 (42%)	192 (46%)	393 (40%)	960 (42%)	379 (42%)	168 (41%)	412 (42%)
≥ 100'000	435 (19%)	202 (22%)	91 (22%)	142 (14%)	440 (19%)	172 (19%)	75 (19%)	193 (19%)
Days from last viral load to switch	76 (36, 139)	65 (33, 117)	59 (30, 110)	91 (52, 174)	73 (37, 133)	65 (35, 117)	59 (32, 112)	87 (49, 168)
Days with viremia ≥1000 copies/mL in all consecutive measurements	371 (236, 581)	363 (230, 579)	342 (224, 563)	391 (254, 595)	377 (238, 594)	378 (235, 604)	351 (225, 563)	380 (251, 597)
Any prior visit missed by ≥90 d	624 (27%)	243 (27%)	117 (28%)	264 (27%)	619 (27%)	240 (26%)	111 (27%)	268 (27%)
Quarter of switch								
Q4 2019/Q1 2020	427 (18%)	318 (35%)	23 (5.5%)	86 (8.7%)	454 (20%)	176 (19%)	69 (17%)	209 (21%)
Q2 2020	614 (26%)	225 (25%)	88 (21%)	301 (30%)	485 (21%)	194 (21%)	95 (23%)	196 (20%)
Q3 2020	543 (23%)	164 (18%)	99 (24%)	280 (28%)	478 (21%)	190 (21%)	89 (22%)	199 (20%)
Q4 2020	294 (13%)	94 (10%)	82 (20%)	118 (12%)	461 (20%)	178 (20%)	79 (19%)	204 (21%)
Q1/Q2 2021	443 (19%)	114 (12%)	123 (30%)	206 (21%)	429 (19%)	173 (19%)	75 (18%)	182 (18%)
Facility-level retention								
First (lowest) quintile	431 (19%)	171 (19%)	87 (21%)	173 (17%)	424 (18%)	169 (19%)	69 (17%)	186 (19%)
Second quintile	491 (21%)	229 (25%)	93 (22%)	169 (17%)	603 (26%)	239 (26%)	108 (27%)	256 (26%)
Third quintile	479 (21%)	216 (24%)	73 (18%)	190 (19%)	543 (24%)	214 (23%)	97 (24%)	232 (23%)
Fourth quintile	478 (21%)	180 (20%)	54 (13%)	244 (25%)	285 (12%)	112 (12%)	51 (13%)	122 (12%)
Fifth (highest) quintile	442 (19%)	119 (13%)	108 (26%)	215 (22%)	453 (20%)	177 (19%)	81 (20%)	196 (20%)

Categorical variables are shown as *n* (%) and continuous variables as median (IQR).

Abbreviations: ART, antiretroviral therapy; AZT, zidovudine; DTG, dolutegravir; IPTW, inverse probability of treatment weighting; LPV/r, ritonavir-boosted lopinavir; TDF, tenofovir disoproxil fumarate; XTC, lamivudine or emtricitabine.

^a^Closest CD4 cell count to ART initiation within ≤180 d before to ≤30 d after ART initiation.

^b^Most recent CD4 cell count before switch to second-line ART and >30 d after ART initiation; missing category included in models.

### Death and LTFU

In the outcome cohort, the crude proportion of death or LTFU was 283/915 (31%) with AZT/XTC/LPV/r, 117/415 (28%) with AZT/XTC/DTG, and 320/991 (32%) with TDF/XTC/DTG. This included 3 (0%), 9 (2%), and 17 (2%) deaths, respectively. Facility transfers were recorded for 52/915 (6%) with AZT/XTC/LPV/r, 22/415 (5%) with AZT/XTC/DTG, and 52/991 (5%) with TDF/XTC/DTG. Regimen changes were recorded for 200/915 (22%) in the AZT/XTC/LPV/r, 95/415 (23%) in the AZT/XTC/DTG, and 147/991 (15%) in the TDF/XTC/DTG group (Table 3).

**Table 3.** Crude Outcomes

Outcome	Overall *N* = 2321	AZT/XTC/LPV/r *N* = 915	AZT/XTC/DTG *N* = 415	TDF/XTC/DTG *N* = 991
Retention outcomes, *n* (%)				
Death or LTFU	720 (31%)	283 (31%)	117 (28%)	320 (32%)
Death	29 (1%)	3 (0%)	9 (2%)	17 (2%)
LTFU	691 (30%)	280 (31%)	108 (26%)	303 (31%)
Days to death or LTFU, median (IQR)	317 (126, 508)	310 (115, 525)	342 (189, 524)	293 (111, 482)
Transfer-out^a^	126 (5%)	52 (6%)	22 (5%)	52 (5%)
Days to transfer-out, median (IQR)	291 (141, 513)	316 (153, 556)	420 (146, 528)	197 (140, 276)
In care without 24-m VL	205 (9%)	59 (6%)	36 (9%)	110 (11%)
In care with 24-m VL	1270 (55%)	521 (57%)	240 (58%)	509 (51%)
Days to VL result, median (IQR)	727 (668, 787)	731 (679, 795)	721 (668, 780)	722 (653, 783)
Regimen change, *n* (%)	442 (19%)	200 (22%)	95 (23%)	147 (15%)
Days to regimen change, median (IQR)	251 (111, 497)	376 (156, 555)	251 (126, 474)	182 (84, 337)
VL outcomes, *n* (%)^b^	*N* = 1270	*N* = 521	*N* = 240	*N* = 509
≤50 copies/mL	739 (58%)	272 (52%)	146 (61%)	321 (63%)
51–999 copies/mL	273 (21%)	107 (21%)	56 (23%)	110 (22%)
≥1000 copies/mL	258 (20%)	142 (27%)	38 (16%)	78 (15%)

Abbreviations: AZT, zidovudine; CI, confidence interval; DTG, dolutegravir; IQR, interquartile range; LTFU, loss to follow-up; TDF, tenofovir disoproxil fumarate; VL, viral load; XTC, emtricitabine or lamivudine.

^a^16 thereof (8 initially switched to AZT/XTC/LPV/r, 4 to AZT/XTC/DTG, and 4 to TDF/XTC/DTG) transferred out after a regimen change.

^b^Among those who remained in care with a 24-m VL result.

In intention-to-treat analysis, the standardized 24-month risk of death or LTFU was similar between regimens at 31% (95% CI: 27%, 34%) with AZT/XTC/LPV/r, 30% (95% CI: 24%, 35%) with AZT/XTC/DTG (aRD to AZT/XTC/LPV/r: −1% [95% CI: −8%, 6%]), and 34% (95% CI: 30%, 37%) with TDF/XTC/DTG (aRD to AZT/XTC/LPV/r: 3% [95% CI: −2%, 8%]; aRD to AZT/XTC/DTG: 4% [95% CI: −2%, 11%]; Table 4).

**Table 4.** Coprimary Endpoints and Sensitivity Analyses

Outcome	AZT/XTC/LPV/r	AZT/XTC/DTG	TDF/XTC/DTG	ARD, AZT/XTC/DTG -AZT/XTC/LPV/r	ARD, TDF/XTC/DTG -AZT/XTC/LPV/r	ARD, TDF/XTC/DTG—AZT/XTC/DTG
Coprimary endpoints, intention-to-treat analyses, % (95% CI)						
Standardized risk of death or LTFU	31% (27%, 34%)	30% (24%, 35%)	34% (30%, 37%)	−1% (−8%, 6%)	3% (−2%, 8%)	4% (−2%, 11%)
Standardized risk of viremia >50 copies/mL	50% (45%, 55%)	40% (32%, 47%)	39% (34%, 44%)	−10% (−19%, −2%)	−11% (−18%, −5%)	−1% (−9%, 8%)
Coprimary endpoints, per-protocol analyses, % (95% CI)						
Standardized risk of death or LTFU	31% (28%, 35%)	27% (21%, 33%)	33% (30%, 37%)	−4% (−11%, 3%)	2% (−3%, 7%)	6% (−1%, 13%)
Standardized risk of viremia >50 copies/mL	51% (46%, 57%)	39% (31%, 47%)	39% (32%, 43%)	−12% (−22%, −3%)	−12% (−21%, −7%)	0% (−10%, 8%)

Standardized risks of loss death or loss to follow-up were calculated from monthly hazard ratios estimated using weighted pooled logistic regression models. Standardized risks of viremia were calculated with weighted logistic regression models.

Abbreviations: aRD, adjusted risk difference; AZT, zidovudine; CI, confidence interval; DTG, dolutegravir; LTFU, loss to follow-up; TDF, tenofovir disoproxil fumarate; VL, viral load; XTC, emtricitabine or lamivudine

In per-protocol analysis, standardized risks were similar at 31% (95% CI: 28%, 35%) with AZT/XTC/LPV/r, 27% (95% CI: 21%, 33%) with AZT/XTC/DTG (aRD to AZT/XTC/LPV/r: −4% [95% CI: −11%, 3%), and 33% (95% CI: 30%, 37%) with TDF/XTC/DTG (aRD to AZT/XTC/LPV/r: 2% [95% CI: −3%, 7%]; aRD to AZT/XTC/DTG: 6% [95% CI: −1%, 13%]; Table 4).

### Viremia

A 24-month viral load result was available for 521/915 (57%) in the AZT/XTC/LPV/r group, 240/415 (58%) in the AZT/XTC/DTG group, and 509/991 (51%) in the TDF/XTC/DTG group. This corresponds to 521/580 (90%), 240/276 (87%), and 509/619 (82%), respectively, of those retained in care at 24 months. The median time from baseline to the 24-month viral load result was 727 days (IQR: 668, 787) and was similar between groups. Among available 24-month viral loads, 249/521 (48%) in the AZT/XTC/LPV/r group, 94/240 (39%) in the AZT/XTC/DTG group, and 188/509 (37%) in the TDF/XTC/DTG group were viremic (Table 3). Findings were similar when considering all available viral load results in the 24-month outcome window (Supplementary Table 4).

In intention-to-treat analysis, the standardized risk of viremia at 24 months among people retained in care with a 24-month viral load result was higher with AZT/XTC/LPV/r (50% [95% CI: 45%, 55%]) than with AZT/XTC/DTG (40% [95% CI: 32%, 47%]; aRD: −10% [95% CI: −19%, −2%]) or TDF/XTC/DTG (39% [95% CI: 34%, 44%]; aRD: −11% [95% CI: −18%, −5%]), but similar between AZT/XTC/DTG and TDF/XTC/DTG (aRD: −1% [95% CI: −9%, 8%]; Table 4).

Similarly, in per-protocol analysis the standardized risk was 51% (95% CI: 46%, 57%) in the AZT/XTC/LPV/r group, 39% (95% CI: 31%, 47%) in the AZT/XTC/DTG group (aRD to AZT/XTC/LPV/r: −12% [95% CI: −22%, −3%]), and 39% (95% CI 32%, 43%) in the TDF/XTC/DTG group (aRD to AZT/XTC/LPV/r: −12% [95% CI: −21%, −7%]; aRD to AZT/XTC/DTG: 0% [95% CI: −10%, 8%; Table 4).

### Sensitivity Analyses

The standardized risk for the composite outcome of risk of death, LTFU, or transfer-out gave similar results to the coprimary endpoint censoring at transfer-out in both intention-to-treat and per-protocol analysis (Supplementary Table 5).

Defining viremia at a threshold of ≥1000 copies/mL, the crude proportion of viremia among those retained in care with a 24-month viral load result was 142/521 (27%) in the AZT/XTC/LPV/r group, 38/240 (16%) with AZT/XTC/DTG, and 78/509 (15%) with TDF/XTC/DTG (Table 3). In line with the coprimary endpoint of viremia >50 copies/mL, the standardized risk of viremia ≥1000 copies/mL was higher with AZT/XTC/LPV/r than with AZT/XTC/DTG or TDF/XTC/DTG and similar between AZT/XTC/DTG and TDF/XTC/DTG in both intention-to-treat and per-protocol analysis (Supplementary Table 5).

## DISCUSSION

In this large cohort study with target trial emulation, we observed differential second-line regimen uptake by gender, similar 24-month risk of death or LTFU with different second-line regimens, and lower 24-month viremia with AZT/XTC/DTG and TDF/XTC/DTG compared with AZT/XTC/LPV/r.

As we only considered switches from TDF/XTC/EFV, the number of switches declined over time with progressing phase-out of EFV- in favor of DTG-based first-line ART. Within first-line ART, we previously showed that women were less likely than men both to newly initiate DTG- compared with EFV-based first-line ART, and to transition from EFV- to DTG-based first-line ART, in the early stages of the DTG rollout in South Africa [6]. In the present study, the lower probability of women to receive a DTG-based regimen upon treatment switch was driven by the early phase of the DTG rollout and subsequently equalized with updated guidance recommending DTG for all.

Previous randomized trials have compared DTG and LPV/r, or DTG with a backbone containing TDF or AZT, in second-line ART. The DAWNING trial (*N* = 624) assessed 48-week viral suppression to <50 copies/mL among adults randomized to second-line DTG or LPV/r, each alongside an NRTI backbone with at least one fully active NRTI based on genotypic resistance testing. At 84% in the DTG and 70% in the LPV/r group, the trial concluded superiority of DTG [3]. In children and adolescents, the ODYSSEY trial compared first- and second-line DTG-based ART with the respective standard of care. In the second-line group (*N* = 396), the NRTI backbone was designed to include at least one NRTI with preserved activity based on resistance testing or assumed based on treatment history, and the standard of care mostly consisted of LPV/r-based ART. At 96-weeks, viral suppression to <50 copies/mL was achieved by 81% receiving DTG and 72% with the standard of care [20]. Finally, the NADIA trial randomized participants failing first-line ART to either DTG or darunavir as the second-line core agent, and to either recycling tenofovir/lamivudine from first-line ART or changing to AZT/lamivudine as the second-line NRTI backbone. Among those receiving DTG (*N* = 235), 84% with recycled tenofovir/lamivudine versus 77% changing to AZT/lamivudine had 96-week viral suppression to <50 copies/mL, indicating noninferiority [5]. To our knowledge, no randomized trial has directly compared switching to LPV/r- versus DTG-based second-line ART among adults beyond 48 weeks.

Our findings thus largely align with available evidence from randomized trials (although with far higher proportions of viremia in this routine care cohort) and with a previous assessment of 12-month second-line treatment outcomes in this setting [7]. Our findings are encouraging regarding the continued rollout of DTG and recycling of the TDF/XTC backbone from first-line ART, but also highlight substantial gaps in retention in care and viral suppression. This is of particular concern in light of emergent DTG resistance [8–10] and considering even low-level viremia below 1000 copies/mL, which here make up more than half of recorded viremia, is associated with subsequent higher-level viremia and/or treatment failure [21–23]. Better strategies to improve retention in care and support adherence are thus urgently needed to prevent failure of second-line ART. In addition, increased access to resistance testing and third-line treatment may be needed for adequate management of second-line treatment failure.

The strengths of this study include its large sample size, near-complete coverage of eligible switches in the study population, 24-month follow-up, representation of treatment outcomes in routine care, and use of target trial emulation methodology aiming to minimize bias. However, it also has limitations. First, the study period coincides with the transition from TDF/XTC/EFV to TDF/XTC/DTG in first-line ART. This could disadvantage the TDF/XTC/DTG group in 2 ways: people taking TDF/XTC/EFV whose viremia was not appropriately addressed might have later changed to TDF/XTC/DTG within the first-line transition without adequate adherence support (we do not have data on provision of enhanced adherence counseling), whereas switches to the other 2 regimens indicate deliberate clinical action; furthermore, for people with treatment failure who received enhanced adherence counseling while taking TDF/XTC/EFV but had clear adherence challenges, the switch to second-line ART may have been delayed with the aim to address adherence first until they were switched to TDF/XTC/DTG by default with the progressing phasing-out of EFV. Both hypotheses are supported by the longer time from treatment failure to switch in the TDF/XTC/DTG group. These considerations might explain the relatively high point estimate for death or LTFU with TDF/XTC/DTG and make the observed similar retention and superior viral suppression compared with AZT/XTC/LPV/r all the more reassuring. Second, we were not able to link individuals between healthcare facilities but instead relied on TIER.Net patient outcome reporting for transfers and death, and then derived LTFU from unexplained missed clinic visits. This may have led to an overestimation of LTFU and underestimation of death and transfers [18]. Third, while target trial emulation aims to minimize bias, we cannot rule out residual unmeasured confounding.

Overall, our findings suggest similar retention in care and superior viral suppression with DTG-based second-line regimens, compared with AZT/XTC/LPV/r. However, they also highlight the urgent need to improve treatment outcomes of second-line ART. Overall, these findings support the WHO recommendation for preferred use of DTG-based second-line ART.

## Supplementary Material

ofaf530_Supplementary_Data

## Supplementary Data

Supplementary materials are available at *Open Forum Infectious Diseases* online. Consisting of data provided by the authors to benefit the reader, the posted materials are not copyedited and are the sole responsibility of the authors, so questions or comments should be addressed to the corresponding author.

## References

[1] World Health Organization . Interim guidelines: updated recommendations on first-line and second-line antiretroviral regimens and post-exposure prophylaxis and recommendations on early infant diagnosis of HIV. Geneva, Switzerland: World Health Organization, 2018. Available at: https://apps.who.int/iris/bitstream/handle/10665/277395/WHO-CDS-HIV-18.51-eng.pdf?ua=1. Accessed 16 August 2022.

[2] World Health Organization . Policy brief. Updated recommendations on first-line and second-line antiretroviral regimens and post-exposure prophylaxis and recommendations on early infant diagnosis of HIV. Geneva, Switzerland: World Health Organization, 2018. Available at: https://iris.who.int/bitstream/handle/10665/273632/WHO-CDS-HIV-18.18-eng.pdf?ua=1. Accessed 16 August 2022.

[3] Aboud M, Kaplan R, Lombaard J, et al Dolutegravir versus ritonavir-boosted lopinavir both with dual nucleoside reverse transcriptase inhibitor therapy in adults with HIV-1 infection in whom first-line therapy has failed (DAWNING): an open-label, non-inferiority, phase 3b trial. Lancet Infect Dis 2019; 19:253–64. 30732940 10.1016/S1473-3099(19)30036-2

[4] Paton NI, Musaazi J, Kityo C, et al Dolutegravir or darunavir in combination with zidovudine or tenofovir to treat HIV. N Engl J Med 2021; 385:330–41. 34289276 10.1056/NEJMoa2101609

[5] Paton NI, Musaazi J, Kityo C, et al Efficacy and safety of dolutegravir or darunavir in combination with lamivudine plus either zidovudine or tenofovir for second-line treatment of HIV infection (NADIA): week 96 results from a prospective, multicentre, open-label, factorial, randomised, non-inferiority trial. Lancet HIV 2022; 9:e381–93. 35460601 10.1016/S2352-3018(22)00092-3

[6] Dorward J, Sookrajh Y, Khubone T, et al Implementation and outcomes of dolutegravir-based first-line antiretroviral therapy for people with HIV in South Africa: a retrospective cohort study. Lancet HIV 2023; 10:e284–94. 37001536 10.1016/S2352-3018(23)00047-4 PMC10288006

[7] Asare K, Sookrajh Y, van der Molen J, et al Clinical outcomes with second-line dolutegravir in people with virological failure on first-line non-nucleoside reverse transcriptase inhibitor-based regimens in South Africa: a retrospective cohort study. Lancet Glob Health 2024; 12:e282–91. 38142692 10.1016/S2214-109X(23)00516-8 PMC10805003

[8] Loosli T, Hossmann S, Ingle SM, et al HIV-1 drug resistance in people on dolutegravir-based antiretroviral therapy: a collaborative cohort analysis. Lancet HIV 2023; 10:e733–41. 37832567 10.1016/S2352-3018(23)00228-X PMC10913014

[9] Tschumi N, Lukau B, Tlali K, et al Emergence of acquired dolutegravir resistance in treatment-experienced people with HIV in Lesotho. Clin Infect Dis 2024; 79:1208–22. 38567806 10.1093/cid/ciae185 PMC11581688

[10] Skrivankova VW, Huwa J, Muula G, et al Virologic failure and drug resistance after programmatic switching to dolutegravir-based first-line antiretroviral therapy in Malawi and Zambia. Clin Infect Dis 2025; 80:120–8. 38847281 10.1093/cid/ciae261 PMC11797054

[11] Hansford HJ, Cashin AG, Jones MD, et al Reporting of observational studies explicitly aiming to emulate randomized trials: a systematic review. JAMA Netw Open 2023; 6:e2336023. 37755828 10.1001/jamanetworkopen.2023.36023 PMC10534275

[12] Hernán MA, Robins JM. Using big data to emulate a target trial when a randomized trial is not available. Am J Epidemiol 2016; 183:758–64. 26994063 10.1093/aje/kwv254 PMC4832051

[13] von Elm E, Altman DG, Egger M, Pocock SJ, Gøtzsche PC, Vandenbroucke JP. The strengthening the reporting of observational studies in epidemiology (STROBE) statement: guidelines for reporting observational studies. Lancet 2007; 370:1453–7. 18064739 10.1016/S0140-6736(07)61602-X

[14] Simon-Tillaux N, Martin GL, Hajage D, et al Conducting observational analyses with the target trial emulation approach: a methodological systematic review. BMJ Open 2024; 14:e086595. 10.1136/bmjopen-2024-086595 PMC11574403 39532374

[15] Hansford HJ, Cashin AG, Jones MD, et al Development of the TrAnsparent ReportinG of observational studies emulating a target trial (TARGET) guideline. BMJ Open 2023; 13:e074626. 10.1136/bmjopen-2023-074626 PMC10503363 37699620

[16] Zuo H, Yu L, Campbell SM, Yamamoto SS, Yuan Y. The implementation of target trial emulation for causal inference: a scoping review. J Clin Epidemiol 2023; 162:29–37. 37562726 10.1016/j.jclinepi.2023.08.003

[17] Osler M, Hilderbrand K, Hennessey C, et al A three-tier framework for monitoring antiretroviral therapy in high HIV burden settings. J Int AIDS Soc 2014; 17:18908. 24780511 10.7448/IAS.17.1.18908 PMC4005043

[18] Etoori D, Wringe A, Kabudula CW, et al Misreporting of patient outcomes in the South African national HIV treatment database: consequences for programme planning, monitoring, and evaluation. Front Public Health 2020; 8:100. 32318534 10.3389/fpubh.2020.00100 PMC7154050

[19] Cole SR, Hernán MA. Constructing inverse probability weights for marginal structural models. Am J Epidemiol 2008; 168:656–64. 18682488 10.1093/aje/kwn164 PMC2732954

[20] Turkova A, White E, Mujuru HA, et al Dolutegravir as first- or second-line treatment for HIV-1 infection in children. N Engl J Med 2021; 385:2531–43. 34965338 10.1056/NEJMoa2108793 PMC7614690

[21] Bareng OT, Moyo S, Mudanga M, et al Low-level viremia among adults living with HIV on dolutegravir-based first-line antiretroviral therapy is a predictor of virological failure in Botswana. Viruses 2024; 16:720. 38793602 10.3390/v16050720 PMC11125697

[22] Kohler M, Brown JA, Tschumi N, et al Clinical relevance of human immunodeficiency virus low-level viremia in the dolutegravir era: data from the Viral Load Cohort North-East Lesotho (VICONEL). Open Forum Infect Dis 2024; 11:ofae013. 38390465 10.1093/ofid/ofae013 PMC10883284

[23] Sodeke O, Milligan K, Ezeuko I, et al Longitudinal viral load outcomes of adults with HIV after detectable viremia on tenofovir, lamivudine, and dolutegravir. AIDS 2024; 38:1714–9. 38870005 10.1097/QAD.0000000000003956 PMC11293980

