# Supplementary material for: Uptake and 24-month Outcomes of Dolutegravir- Versus Lopinavir-based Second-line Antiretroviral Therapy for People With HIV in South Africa: A Retrospective Cohort Study and Emulated Target Trial
Source: Open Forum Infect Dis. 2025 Aug 30;12(9):ofaf530. doi: 10.1093/ofid/ofaf530 (PMC12448401; doi:10.1093/ofid/ofaf530)
Supplement: ofaf530_Supplementary_Data [file ofaf530_supplementary_data.docx]

# Supplementary material

# Uptake and 24-month outcomes of dolutegravir- versus lopinavir-based second-line antiretroviral therapy for people with HIV in South Africa: a retrospective cohort study and emulated target trial


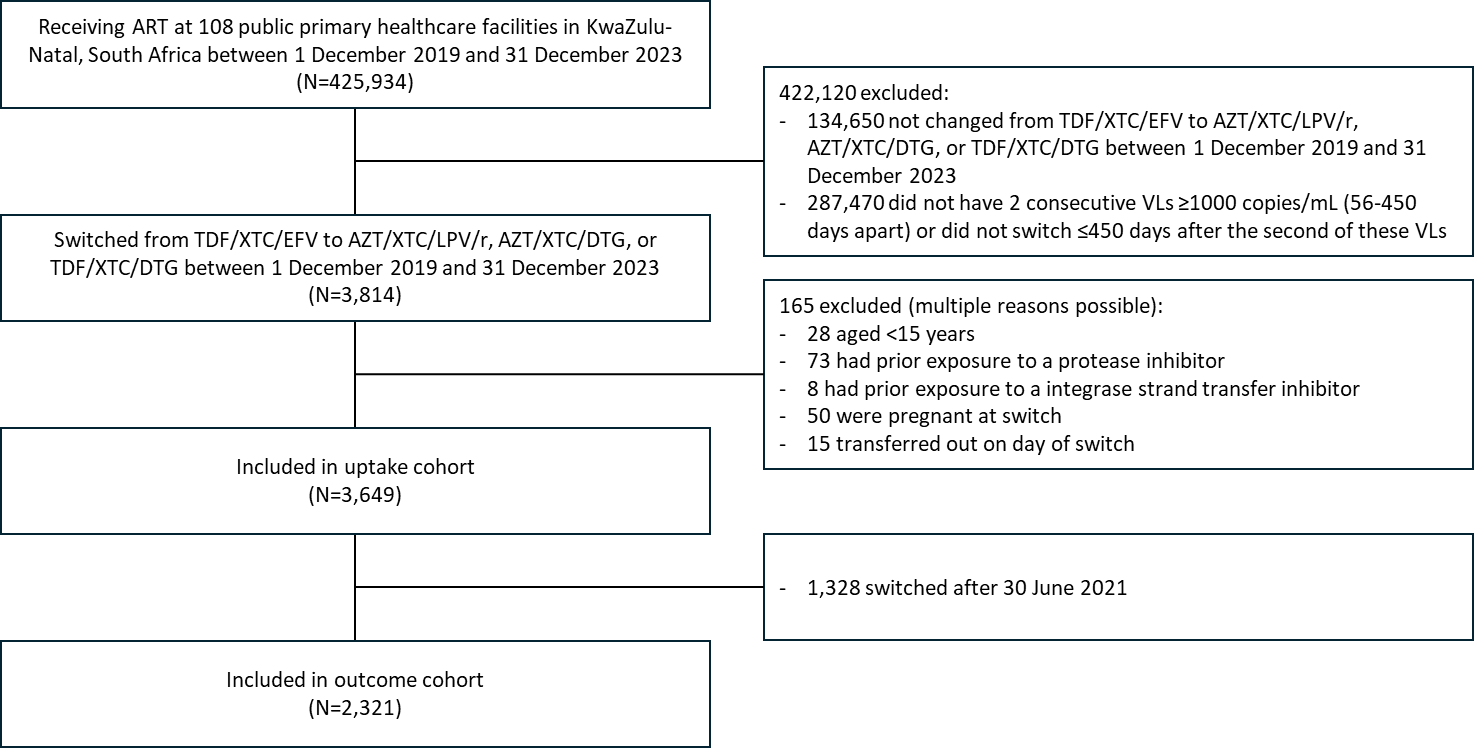


## Supplementary Figure 1: Flow diagram. ART: antiretroviral therapy; AZT: zidovudine; DTG: dolutegravir; EFV: efavirenz; LPV/r: ritonavir-boosted lopinavir; TDF: tenofovir disoproxil fumarate; VL: viral load; XTC: lamivudine or emtricitabine.

**Alt text:** Flow diagram showing that among 425,934 people receiving ART in an included primary healthcare facility during the study period, 422,120 were excluded due to not having an eligible regimen switch and a further 165 met other exclusion criteria. The remaining 3,649 were included in the uptake cohort, and of these, 2,321 were included in the outcome cohort.

## Supplementary Table 1: Specification of the target trial and emulated trial. ART: antiretroviral therapy; AZT: zidovudine; DTG: dolutegravir; EFV: efavirenz; LPV/r: ritonavir-boosted lopinavir; LTFU: loss to follow-up; TDF: tenofovir disoproxil fumarate; XTC: lamivudine or emtricitabine.

| **Protocol** | **Target trial** | **Emulated trial** |
| --- | --- | --- |
| Eligibility criteria | Taking TDF/XTC/EFV and due to switch to TDF/XTC/DTG, AZT/XTC/DTG, or AZT/XTC/LPV/r | Switched from TDF/XTC/EFV to TDF/XTC/DTG, AZT/XTC/DTG, or AZT/XTC/LPV/r |
|  | Confirmed virological failure, defined as two consecutive viral loads ≥1000 copies/mL and taken 56-450 days apart (intercurrent viral loads within 56 days are permitted if they are also ≥1000 copies/mL), with the latter occurring ≤450 days before enrolment | Switch occurred after confirmed virological failure, defined as two consecutive viral loads ≥1000 copies/mL and taken 56-450 days apart (intercurrent viral loads within 56 days are permitted if they are also ≥1000 copies/mL), with the latter occurring ≤450 days before the switch |
|  | Eligibility to switch during study period | Switch occurred between 1 December 2019 (start of availability of DTG) and 30 June 2021 (allowing 30 months’ of follow-up until data closure on 31 December 2023) |
|  | No known prior PI or INSTI exposure | Same |
|  | Age ≥15 years at switch | Same |
|  | Not pregnant at switch | Same |
|  | In care in a study site | In care in a facility with available data through 31 December 2023 |
| Treatment strategies | Switch to TDF/XTC/DTG | Same |
|  | Switch to AZT/XTC/DTG | Same |
|  | Switch to AZT/XTC/LPV/r | Same |
| Treatment assignment | Participants are randomly assigned and are aware of the strategy they are assigned to (open-label) | Randomisation is assumed conditional on baseline covariates used in inverse probability weighting^a^. |
| Follow-up period | 24 months for death and LTFU outcomes and 30 months for viral load outcome. | Same |
| Outcomes: primary endpoints | Death or LTFU through 24 months. | Same. The date of LTFU is defined as the midpoint between the last attended visit and the first visit missed by ≥90 days^b^. |
|  | 24-month viraemia, defined as having a viral load >50 copies/mL among those retained in care and with a 24-month viral load result (window: 18-30 months; using the closest viral load to 24 months). | Same |
| Outcomes: sensitivity analysis | Death, LTFU, or transfer-out through 24 months. | Same |
|  | 24-month viraemia, defined as having a viral load ≥1000 copies/mL among those retained in care and with a 24-month viral load result (window: 18-30 months; using the closest viral load to 24 months). | Same |
| Causal contrasts | Intention-to-treat analysis. Participants are censored upon transfer-out (except for the sensitivity analysis with transfer out as an endpoint). | Observational analogue thereof |
|  | Per-protocol analysis. Participants are censored upon transfer-out (except for the sensitivity analysis with transfer out as an endpoint) or regimen change. | Observational analogue thereof |
| Statistical analysis | In intention to treat analysis, death or LTFU is assessed through pooled logistic regression. Viraemia is assessed through logistic regression among people retained in care with a 24-month viral load result. | Same with inverse probability of treatment weighting as described above to emulate randomisation. |
|  | In per-protocol analysis, death or LTFU is assessed through pooled logistic regression weighted for inverse probability of remaining uncensored (weights derived using baseline^a^ and time-varying^c^ covariates).  Viraemia is assessed through logistic regression among people retained in care with a 24-month viral load result, weighted for inverse probability of remaining uncensored at 24 months (assessed with baseline^a^ and time-varying^c^ covariates) as well as inverse probability of having a 24-month viral load measurement (weights derived using baseline covariates^a^). | Same with additional inverse probability of treatment weighting as described above to emulate randomisation. |

^a^ Categorical baseline covariates: gender, age, calendar time of switch, region, prior decentralised ART, last viral load before switch, last CD4 cell count before switch, previously having missed a visit by ≥90 days, facility retention category (proportion of people in care in the facility with a missed visit in the year prior to the start of study enrolment; facilities categorised in quintiles). Continuous baseline covariates: days since first consecutive viral load ≥1000 copies/mL.

^b^ Different intervals between clinic visits (generally ranging from one to six months) could bias outcomes if the date of LTFU were set as the date of the last attended or first missed visit. To account for this, the date of LTFU is defined as the midpoint between the last attended visit and the first visit missed by ≥90 days, and the endpoint is reached if this LTFU date is within 24 months of switching. With maximal visit intervals of six months, the missed visit would have to be scheduled within 3 months of the end of follow-up for the LTFU date to fall within the 24 months’ follow-up period. Ascertainment of whether this visit was missed would require an additional 90 days. Thus, 30 months between switch and data closure are sufficient to ascertain 24-month LTFU.

^c^ Categorical time-varying covariates: pregnancy, tuberculosis, receiving decentralised ART (less-frequent clinic visits). Continuous time-varying covariates: months since switch, square of months since switch.

## Supplementary Table 2: Models of dolutegravir uptake by gender.

| **Model** | **R code^a^** |
| --- | --- |
| *Uptake model 1:* |  |
| Gender only | glm(  dtg ~ gender,  family = poisson(link = "log"),  data = uptake_cohort  ) |
| *Uptake model 2:* |  |
| Gender and age with interaction term | glm(  dtg ~ gender * baseline_age_category,  family = poisson(link = "log"),  data = uptake_cohort  ) |
| Without interaction term (for likelihood ratio test) | glm(  dtg ~ gender + baseline_age_category,  family = poisson(link = "log"),  data = uptake_cohort  ) |
| *Uptake model 3:* |  |
| Gender and calendar period with interaction term | glm(  dtg ~ gender * calendar_period,  family = poisson(link = "log"),  data = uptake_cohort  ) |
| Without interaction term (for likelihood ratio test) | glm(  dtg ~ gender + calendar_period,  family = poisson(link = "log"),  data = uptake_cohort  ) |

^a^ Variable names modified for clarity. Variables: baseline_age_category, age category at baseline (15-29, 30-44, ≥45 years); calendar_period, calendar period of switch (binary, before or after 1 June 2021); dtg, assignment to dolutegravir-based ART regimen (binary); gender, binary gender; uptake_cohort, uptake cohort in wide format (one individual per row).

## Supplementary Table 3: Baseline characteristics in the uptake cohort. Categorical variables are shown as n (%) and continuous variables as median (IQR). ART: antiretroviral therapy; AZT: zidovudine; DTG: dolutegravir; LPV/r: ritonavir-boosted lopinavir; TDF: tenofovir disoproxil fumarate; XTC: lamivudine or emtricitabine.

| **Characteristic** | **Overall** N = 3,649 | **AZT/XTC/LPV/r** N = 998 | **AZT/XTC/DTG** N = 711 | **TDF/XTC/DTG** N = 1,940 |
| --- | --- | --- | --- | --- |
| Women | 2,292 (63%) | 679 (68%) | 435 (61%) | 1,178 (61%) |
| Age in years | 36 (29, 42) | 36 (30, 42) | 36 (30, 42) | 36 (29, 42) |
| 15-29 | 917 (25%) | 240 (24%) | 167 (23%) | 510 (26%) |
| 30-44 | 2,049 (56%) | 587 (59%) | 405 (57%) | 1,057 (54%) |
| ≥45 | 683 (19%) | 171 (17%) | 139 (20%) | 373 (19%) |
| Receiving tuberculosis treatment | 57 (1.6%) | 15 (1.5%) | 17 (2.4%) | 25 (1.3%) |
| Region |  |  |  |  |
| eThekwini Metropolitan Municipality | 2,084 (57%) | 631 (63%) | 432 (61%) | 1,021 (53%) |
| uMgungundlovu District Municipality | 1,565 (43%) | 367 (37%) | 279 (39%) | 919 (47%) |
| Years since ART initiation | 4.0 (2.1, 6.9) | 3.3 (1.7, 6.4) | 4.2 (2.1, 7.2) | 4.3 (2.3, 7.1) |
| CD4 cell count at ART initiation in cells/µL³ ^a^ | 200 (105, 320) | 170 (90, 290) | 170 (90, 300) | 220 (120, 350) |
| <200 | 1,251 (34%) | 410 (41%) | 263 (37%) | 578 (30%) |
| 200-349 | 795 (22%) | 199 (20%) | 132 (19%) | 464 (24%) |
| 350-499 | 343 (9.4%) | 81 (8.1%) | 61 (8.6%) | 201 (10%) |
| ≥500 | 219 (6.0%) | 43 (4.3%) | 26 (3.7%) | 150 (7.7%) |
| Missing | 1,041 (29%) | 265 (27%) | 229 (32%) | 547 (28%) |
| Days from CD4 count at ART initiation to ART initiation | 0 (0, 0) | 0 (0, 0) | 0 (0, 0) | 0 (0, 0) |
| Missing | 1,041 (29%) | 265 (27%) | 229 (32%) | 547 (28%) |
| Most recent CD4 cell count in cells/µL³ ^b^ | 280 (160, 450) | 250 (130, 420) | 250 (120, 400) | 300 (170, 470) |
| <200 | 834 (23%) | 250 (25%) | 198 (28%) | 386 (20%) |
| 200-349 | 707 (19%) | 181 (18%) | 149 (21%) | 377 (19%) |
| 350-499 | 487 (13%) | 130 (13%) | 88 (12%) | 269 (14%) |
| ≥500 | 509 (14%) | 110 (11%) | 89 (13%) | 310 (16%) |
| Missing | 1,112 (30%) | 327 (33%) | 187 (26%) | 598 (31%) |
| Days from most recent CD4 result to switch | 279 (74, 693) | 241 (61, 635) | 210 (55, 589) | 314 (112, 792) |
| Missing | 1,112 (30%) | 327 (33%) | 187 (26%) | 598 (31%) |
| Prior exposure to AZT | 35 (1.0%) | 6 (0.6%) | 12 (1.7%) | 17 (0.9%) |
| Prior referral to decentralised ART | 775 (21%) | 170 (17%) | 151 (21%) | 454 (23%) |
| Last viral load in copies/mL |  |  |  |  |
| 1'000-9'999 | 1,533 (42%) | 367 (37%) | 252 (35%) | 914 (47%) |
| 10'000-99'999 | 1,481 (41%) | 415 (42%) | 312 (44%) | 754 (39%) |
| ≥100'000 | 635 (17%) | 216 (22%) | 147 (21%) | 272 (14%) |
| Days from last viral load to switch | 84 (42, 156) | 67 (35, 120) | 61 (31, 113) | 100 (56, 188) |
| Days with viraemia ≥1000 copies/mL in all consecutive measurements | 397 (251, 650) | 371 (231, 604) | 364 (235, 587) | 426 (273, 688) |
| Any prior visit missed by ≥90 days | 1,331 (36%) | 283 (28%) | 264 (37%) | 784 (40%) |
| Year of switch |  |  |  |  |
| 2019/2020 | 1,878 (51%) | 801 (80%) | 292 (41%) | 785 (40%) |
| 2021 | 782 (21%) | 157 (16%) | 246 (35%) | 379 (20%) |
| 2022 | 607 (17%) | 31 (3.1%) | 146 (21%) | 430 (22%) |
| 2023 | 382 (10%) | 9 (0.9%) | 27 (3.8%) | 346 (18%) |
| Facility-level retention |  |  |  |  |
| First (lowest) quintile | 686 (19%) | 188 (19%) | 144 (20%) | 354 (18%) |
| Second quintile | 766 (21%) | 252 (25%) | 155 (22%) | 359 (19%) |
| Third quintile | 780 (21%) | 231 (23%) | 151 (21%) | 398 (21%) |
| Fourth quintile | 705 (19%) | 197 (20%) | 103 (14%) | 405 (21%) |
| Fifth (highest) quintile | 712 (20%) | 130 (13%) | 158 (22%) | 424 (22%) |

^a^ Closest CD4 cell count to ART initiation within ≤180 days before to ≤30 days after ART initiation.

^b^ Most recent CD4 cell count before switch to second-line ART and >30 days after ART initiation.

## Supplementary Table 4: Viral load results among participants with at least one VL in the outcome window. AZT: zidovudine; DTG: dolutegravir; LPV/r: ritonavir-boosted lopinavir; TDF: tenofovir disoproxil fumarate; XTC: lamivudine or emtricitabine.

| **VL outcomes** | **Overall**  N=1270 | **AZT/XTC/LPV/r**  N=521 | **AZT/XTC/DTG**  N=240 | **TDF/XTC/DTG**  N=509 |
| --- | --- | --- | --- | --- |
| Number of VLs in outcome window, median (IQR) [range] | 1 (1, 2) [1, 4] | 1 (1, 2) [1, 4] | 1 (1, 2) [1, 4] | 1 (1, 2) [1, 4] |
| Days to first VL in outcome window, median (IQR) | 678 (609, 761) | 679 (615, 764) | 673 (615, 755) | 673 (605, 761) |
| Summary of all VLs in outcome window, n (%) |  |  |  |  |
| All <50 | 649 (51%) | 234 (45%) | 133 (55%) | 282 (55%) |
| Any >=50 | 621 (49%) | 287 (55%) | 107 (45%) | 227 (45%) |

## Supplementary Table 5: Sensitivity analyses. Standardised risks of loss death or loss to follow-up were calculated from monthly hazard ratios estimated using weighted pooled logistic regression models. Standardised risks of viraemia were calculated with weighted logistic regression models. aRD: adjusted risk difference; AZT: zidovudine; CI: confidence interval; DTG: dolutegravir; LTFU: loss to follow-up; TDF: tenofovir disoproxil fumarate; XTC: emtricitabine or lamivudine.

| **Outcome** | **AZT/XTC/LPV/r** | **AZT/XTC/DTG** | **TDF/XTC/DTG** | **aRD,**  **AZT/XTC/DTG -AZT/XTC/LPV/r** | **aRD,**  **TDF/XTC/DTG -AZT/XTC/LPV/r** | **aRD,**  **TDF/XTC/DTG - AZT/XTC/DTG** |
| --- | --- | --- | --- | --- | --- | --- |
| Sensitivity analyses, intention-to-treat, % (95% CI) | | | | | | |
| Risk of death, LTFU, or transfer-out | 36% (32%, 39%) | 33% (29%, 39%) | 38% (35%, 42%) | ‑2% (‑8%, 5%) | 3% (‑2%, 8%) | 5% (‑2%, 11%) |
| Risk of viraemia >1000 copies/mL | 27% (23%, 32%) | 17% (11%, 22%) | 15% (12%, 18%) | ‑10% (‑18%, ‑4%) | ‑12% (‑18%, ‑7%) | ‑2% (‑9%, 5%) |
| Sensitivity analyses, per-protocol, % (95% CI) | | | | | | |
| Risk of death, LTFU, or transfer-out | 35% (32%, 39%) | 30% (25%, 36%) | 38% (35%, 42%) | ‑5% (‑12%, 2%) | 2% (‑4%, 8%) | 7% (1%, 14%) |
| Risk of viraemia >1000 copies/mL | 27% (23%, 33%) | 19% (12%, 27%) | 14% (11%, 17%) | ‑8% (‑18%, ‑1%) | ‑14% (‑20%, ‑8%) | ‑5% (‑14%, 3%) |
